# Feasibility of a 12-month-exercise intervention during and after radiation and chemotherapy in cancer patients: impact on quality of life, peak oxygen consumption, and body composition

**DOI:** 10.1186/s13014-016-0619-5

**Published:** 2016-03-16

**Authors:** Alexander Grabenbauer, Andrea J. Grabenbauer, Rosa Lengenfelder, Gerhard G. Grabenbauer, Luitpold V. Distel

**Affiliations:** Department of Radiation Oncology, Coburg Cancer Center, Coburg, Germany; Fresenius Kabi Deutschland, Bad Homburg, Germany; Department of Radiation Oncology of the University Hospitals and Friedrich-Alexander-University of Erlangen-Nürnberg, Erlangen, Germany

**Keywords:** Cancer patients, Exercise intervention, Physical exercise, Quality of life, Peak oxygen consumption, Body composition

## Abstract

**Background:**

Accumulating evidence suggests that exercise is effective in treating many of the acute and chronic side effects of anti-cancer therapy. A recent meta-analysis supported the use of exercise to prevent or treat fatigue and lymphoedema and to improve functional status in breast cancer patients.

**Patients and methods:**

This trial was intended as a controlled, prospective feasibility study evaluating the impact of physical exercise (PE) in cancer patients during and after treatment with radio- and chemotherapy. Inclusion criteria were previous or ongoing treatment for cancer, motivation for PE of 0.5-1hour duration at least twice weekly for at least 3 months. Continuation of PE was encouraged thereafter. Every three months the following endpoints were assessed: Peak oxygen consumption as measured by supervised cardiopulmonary exercise test, body composition and quality of life.

**Results:**

A total of 45 patients were included with a median age of 49 years. Forty were female and five male. Cancer types were: Breast cancer (*n* = 30/67 %), gastrointestinal cancer (*n* = 5/12 %), other types (*n* = 10/22 %). Thirty-eight (84 %) of the patients were included during curative treatment of their disease. Seven (16 %) were considered palliative. Adherence to the PE-programme longer than 6 months was noted for 41/45 (91 %) of the patients. Intensity of PE was thrice weekly in 32/45 (71 %), twice weekly in 11/45 (24 %). Two of 45 patients (5 %) had no PE. Mean peak oxygen consumption increased from 18.8 ± 5.6 ml/min/kg to 20.5 ± 3 ml/min/kg and 19.9 ± 4.7 ml/min/kg at 3 months (*p* = 0.005) and 12 months (*p* = 0.003), respectively.

Median fat mass decreased from 30.7 ± 15 kg to 28.9 ± 15 kg and 29.5 ± 13 kg at 3 months (*p* = 0.001) and 12 months (*p* = 0.017), respectively. Global health status scores increased from a median baseline value of 54.9 ± 16.3 to 66.4 ± 14 % and 68.0 ± 20.3 % at 3 months (*p* = 0.001) and 12 months (*p* = 0.002), respectively.

**Conclusion:**

This exercise programme in cancer patients with 2–3 weekly supervised sessions over three months was well feasible and demonstrated measurable improvement of oxygen consumption, body composition and quality of life. In addition, a 90 %-adherence rate to the PE-programme beyond 6 months was encouraging. Further randomized prospective data in a larger patient population will be collected comparing the impact of two versus four months supervision.

**Electronic supplementary material:**

The online version of this article (doi:10.1186/s13014-016-0619-5) contains supplementary material, which is available to authorized users.

## Background

Given data from a recent meta-analysis of physical exercise (PE) that indicated a high efficacy in preventing cancer-related fatigue and improving quality of life [1], aerobic supervised PE seems an increasingly fascinating tool. This may prove to be of particular value for patients during and shortly after completion of their cancer treatment since fatigue is usually reported by up to 30–60 % of cancer patients during treatment and up to 25–30 % still complain of fatigue years after treatment [2]. Interestingly, cancer-related fatigue and psychological distress have been identified as predictors for recurrence and survival in breast cancer patients [3].

In addition, PE-programs have been gaining increasing attention even in patients with advanced disease during palliative treatment [4, 5]. Results from these trials indicated a significant increase in muscle strength and aerobic functional fitness as well as improvements in quality of life and social and physical role functioning [4].

When starting a PE-program several issues may need attention including patient selection, patient motivation, and intensity of the aerobic exercise. Of special interest is the selection of the appropriate endpoint of a given study.

This trial was intended as a controlled, prospective feasibility study evaluating the impact of PE in an unselected population of cancer patients during and after treatment. Every three months the endpoints peak oxygen consumption, body composition and quality of life were assessed.

## Patients and methods

This trial was intended as a controlled, prospective feasibility study evaluating the impact of physical exercise (PE) in cancer patients during and after treatment with radio- and chemotherapy. In addition, data on motivation and adherence to a regular PE-programme during a 12-month period should be collected. This trial was approved by the Ethics Committee of the Medical Faculty of the University of Erlangen-Nürnberg under the registration number 4448. All participants provided written, signed informed consent.

### Participant recruitment

**Fig. 1:**
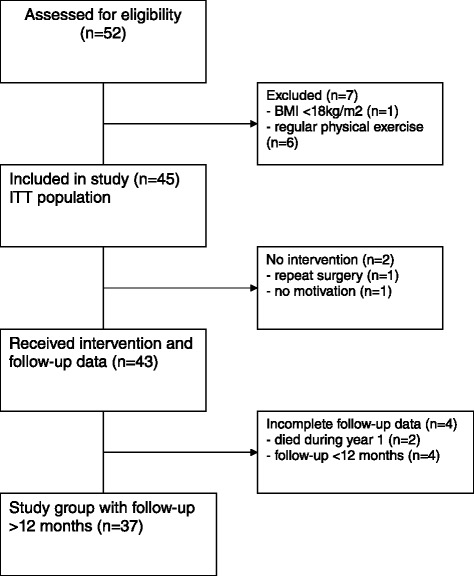
Participant flow diagram

Multiple strategies were used to recruit participants. Patients were primarily recruited through the departments of Radiation Oncology and Gynaecology, were provided with an information sheet by their treating nurses and doctors during routine appointments. In case of an expression of interest by the patient the study nurse explained the study in further detail and checked for eligibility criteria (*n* = 52, Fig. 1). Inclusion criteria were: Age > 18 years, previous or ongoing treatment for cancer, motivation for PE of 0.5–1 hour duration at least twice weekly under supervision of the study nurse for a minimum time of 3 months. Continuation of PE was strongly encouraged thereafter. Exclusion criteria were: body-mass index (BMI) < 18 kg/m2 with no upper limit; performing >60 min/week of moderate vigorous intensity exercise during the previous 6 weeks, and major comorbidities, (e.g. unstable angina, seizures, congestive heart failure of NYHA grade 3–4, uncontrolled infections).

### Exercise intervention

Prior to initiation of the exercise program individual data on oxygen-consumption, optimum aerobic heart-rate and ECG-recordings were performed. Thereafter three weekly individually tailored exercise sessions between 30 and 60 min were done with gradually increasing intensity. During the first three months all sessions were performed at a private gymnasium in small groups under the supervision of the study nurse. The following devices were used depending on individual preferences: Sitzfahrrad Lotus R, Crosstrainer ELYX 3, Coach E Rowing Machine (all manufactured by Kettler, Ense-Parsit, Germany), Treadmill Horizon Ti 52 (Horizon Fitness, New Jersey, USA). All exercise was performed under optimum heart rate as determined by spiroergometry prior to initiation of the program. After that participants were encouraged to continue the exercise program at home and adherence was monitored through participant-completed exercise diaries, regular follow-up visits and/or phone calls.

### Data collection

Demographic characteristics and medical history were obtained from the medical records and by the use of a nurse-driven questionnaire at baseline. All other measures were taken by trained technicians of Department of Cardiology and by the study nurse. Participant recorded any adverse event (bodily or psychological complaints) in their daily exercise diaries regardless of the cause of complaint.

At baseline and every three months peak oxygen consumption (VO_2max_) was quantified by an incremental supervised cardiopulmonary exercise test (CPET). After a 1-min warm-up at 20 W, cycling workload was increased every minute by a predetermined 10, 15, or 20 W until exhaustion or symptom limitation (dyspnea/fatigue). In addition, maximum power (P_max_), and heart rate (HR) at the ventilatory threshold (VT) were determined. According to CPET-guidelines for clinical and cancer populations, a 12-lead electrocardiographic monitoring (VIASYS Healthcare Medizintechnik, Neustadt/A., Germany) was performed during the exercise test. All tests were performed on an electronically braked cycle ergometer (Ergoline ergoselect 200, ergoline, Bitz, Germany) with breath-by-breath expired gas analysis (Erich Jaeger, Höchberg, Germany). The analysis of expired air allowed the determination of oxygen uptake, carbone dioxide production (V_CO2_), ventilation (VE), and respiratory exchange ratio (RER;V_CO2_/V_O2_) during rest and exercise. Maximal oxygen uptake was the highest oxygen uptake during exercise.

Body composition including total body water, fat-free mass, lean body mass, fat mass, body cell mass, extracellular mass, body mass index and phase angle was determined in triplicate by bioelectrical impedance spectroscopy (BIA 2000 S, Medical Health Care GmbH, Karlsruhe) at baseline and monthly during the first three months and every three months thereafter.

Quality of life data were collected using the 30-item Quality of Life Questionaire C-30 [6] as provided by the European Organisation for Research and Treatment of Cancer database.

All statistical data were entered onto PASW statistical software (Version 18; SPSS inc, Chicago, IL), and expressed as mean and median values with standard deviation. The analysis assessed the intervention effect at 3 and 12 months as compared to the values at baseline based on intention-to-treat principles. Baseline-values for all parameters were compared with those at 3 and 12 months using a *t*-test.

The amount of missing data at each assessment was minimal: Two patients died of tumor progression at 9 and 11 months after inclusion in the study, another four patients had incomplete follow-up with missing 12-month-data (Fig. 1). A sample size of 40 patients with a drop-out rate of 5 % was calculated as sufficient to generate early data on feasibility and a possible hypothesis for later investigations

The mean changes in outcomes from baseline to month 3 and 12 were computed by the paired student *t*-test. A normal distribution was noted for all outcomes. Statistical tests were two-sided and p <0.05 was considered statistically significant (Null hypothesis: mean difference is zero and alternative hypothesis: mean difference greater than zero).

## Results

### Recruitment and retention

Of the 52 patients that initially expressed interest, seven did not meet the inclusion criteria: one patient with a critically low body mass index below 18 kg/m^2^ and another six patients that had regular physical exercise of at least 3 h per week. A total of 45 patients provided informed consent and were scheduled for the programme between 06/2011 and 11/2014 (ITT population). Two patients never received the intervention. Reasons were repeated surgery (*n* = 1) and no motivation (*n* = 1) and were therefore excluded for further follow-up. Figure 1 displays the participant flow diagram. A total of 43 patients received the intervention and some follow-up data, of whom four remained with incomplete follow-up data (two patients died during the 12-month- period, and four patients remained with incomplete follow-up of <12 months).

As for the ITT population of 45 patients, patient characteristics are given in Table 1. Median age was 49 years (range, 35–74 years). Forty out of 45 patients (89 %) were female and five (11 %) male. Distribution of cancer types was: Breast cancer (*n* = 30/67 %), gastrointestinal cancer (*n* = 5/12 %), other types (*n* = 10/22 %). Thirty-eight (84 %) of the patients were included during curative treatment of their disease. Seven (16 %) were considered palliative. Ongoing radiation and/or chemotherapy was noted for 37 (82 %) of the patients whereas eight (18 %) of the patients had previous cancer treatments.

**Table 1:** Patients characteristics

Total number of pts		45 (100 %)
Age (years)	Median (Range)	49 (35–74)
Gender	Female	40 (89 %)
	Male	5 (11 %)
Tumor type	Breast Cancer	30 (67 %)
	Colorectal Cancer	5 (11 %)
	Endometrial Cancer	3 (7 %)
	Brain Tumor	3 (7 %)
	Lymphoma	2 (4 %)
	Other	2 (4 %)
Intent of treatment	Curative	38 (84 %)
	Palliative	7 (16 %)
Type of treatment	Ongoing radiation/chemotherapy	37 (82 %)
	Previous treatment	8 (18 %)
Adherence	>3 Months	42 (93 %)
	>6 Months	41 (91 %)
Physical exercise sessions (weekly)	Thrice	32 (71 %)
	Twice	11 (24 %)
	Nil	2 (5 %)

### Adherence

Adherence to the PE-programme for longer than 3 and 6 months was noted for 42/45 (93 %) and 41/45 (91 %) of the patients, respectively. Physical exercise of at least 12 months was completed by 37/45 (82 %) of the patients. Intensity of PE was thrice weekly in 32/45 (71 %), twice weekly in 11/45 (24 %).

### Adverse events

Adverse events attributable to the study included the exacerbation of lumbar spine complaints in one patient, which was later diagnosed as progression of her lymphoma, and anal discomfort with bloody discharge in another patient previously treated for anal cancer. These two patients were advised to discontinue the PE-programme and received treatment for their underlying problems.

### Primary outcomes

Data for body mass composition at baseline and after 1,2,3,6 and 12 months following physical exercise are displayed (Additional file 1: Table S1). As compared with baseline, median body mass index decreased from 27.4 ± 7.2 kg/m^2^ to 25.9 ± 7.0 kg/m^2^ and 26.9 ± 6.7 kg/m^2^ at 3 (*p* = 0.001) and 12 months (*p* = 0.015), respectively. Similar data with decreasing values during the first three months after the intervention were seen for the total fat mass (Additional file 1: Figure S1). Median fat mass decreased from 30.7 ± 15 kg to 28.9 ± 15 kg and 29.5 ± 13 kg at 3 months (*p* = 0.001) and 12 months (*p* = 0.017), respectively. No difference for the body composition-endpoints were detected when comparing the data between 3 and 12 months (*p* = 0.8).

**Fig. 2:**
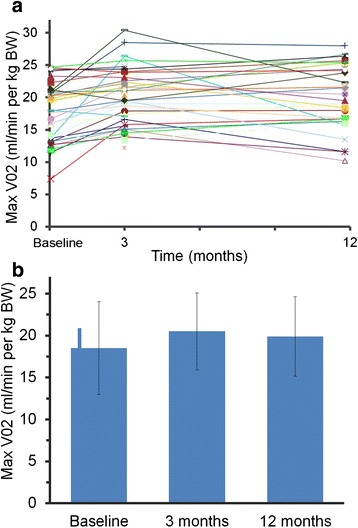
Peak oxygen consumption among 36 cancer patients (**a**) at baseline and after 3 and 12 months (**b**) during continuous physical exercise

Mean peak oxygen consumption increased from 18.8 ± 6 ml/min/kg at baseline to 20.5 ± 5 ml/min/kg and 19.9 ± 5 ml/min/kg at 3 and 12 months, respectively (Fig. 2, Additional file 1: Table S2). The difference between values at baseline vs 3 months (*p* = 0.005) and 12 months (*p* = 0.003) was statistically significant.

**Fig. 3:**
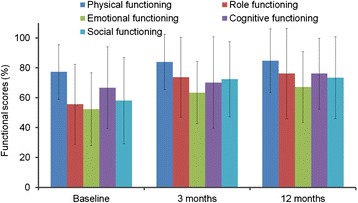
Quality of life data on functional scales for 37 cancer patients at baseline and at 3 and 12 months following continuous physical exercise

Quality of life data on functional scales and symptom scales for all evaluable patients at baseline and at 3 and 12 months following continuous physical exercise are displayed in Fig. 3 and Additional file 1: Figure S2, respectively, as well as in Additional file 1: Tables S3 and S4. Global health status scores increased from a median baseline value of 54.9 ± 16.3 % to 66.4 ± 14 % and 68.0 ± 20.3 % at 3 (*p* < 0.001) and 12 months (*p* = 0.002), respectively (Additional file 1: Figure S3).

### Subgroup analysis

For the endpoint BMI and body fat mass a significant impact was seen comparing patients ≤ 50 years to those of older age. Following aerobic physical exercise BMI decreased by 1.3 ± 1.8 kg/m^2^ only in the group of younger patients at 3 months whereas in older patients the change was only −0.17 ± 1.3 kg/m^2^. This difference reached statistical significance (*p* = 0.03). This difference remained stable at 12 months follow-up (*p* = 0.05) (Additional file 1: Table S5). Changes in body fat mass compared very nicely with BMI-data: Younger patients reached a loss in body fat mass of 2.9 ± 3.5 kg and 3.0 ± 5 kg at 3 and 12 months, as compared to older patients (−0.8 kg and −0.4 kg, *p* = 0.04 and 0.07), respectively (Additional file 1: Table S6). No other factors of significant influence on body mass composition were identified including tumor type (breast cancer vs. others), gender, and treatment intent (palliative with progressive disease vs. curative).

Possible confounders for the endpoints peak oxygen consumption and global health score were also contemplated. Neither age nor gender turned out to have an impact on these endpoints. However, in the subgroup of breast cancer patients VO_2max_ increased by 164 ± 216 ml/min as compared to other patients with 8.9 ± 344 ml/min but did not reach statistical significance (*p* = 0.11) at 3 months. Comparing the group of curative (*n* = 30) vs. palliative patients (*n* = 6), a significant difference in the increase of VO_2max_ was detected (177 ml/min vs. −187 ml/min, *p* = 0.001) at 3 months. At 12 months this difference was no longer seen since only two patients remained for analysis (Additional file 1: Table S7). Comparison of global health score revealed better results for breast cancer patients vs. other patients with an increase of 16.7 ± 17.4 % and 18.7 ± 20.2 % vs. 0.7 ± 16.5 % and −1.7 ± 25.4 % at 3 (*p* = 0.012) and 12 months (*p* = 0.017), respectively (Additional file 1: Table S8).

## Discussion

This prospective trial attempted a supervised exercise intervention in cancer patients during radiation and chemotherapy. It has been the primary intention of this study to provide useful patient data including improvements in oxygen consumption, quality of life and body composition to inform effect size estimation and guide for a possible hypothesis in future large-scale trials.

The first and most important step seemed to motivate patients for a possible participation in the PE-trial. Motivation and preferences for exercise programmes in patients with lung cancer have recently been reported [7]. Among a total of 60 patients a total of 63 % indicated that they had the motivation to exercise. Significant factors associated with motivation to exercise were: being a non-smoker, having a past exercise history prior to diagnosis and absence of COPD. In our experience, the initial patient information by the attending physician and the study nurse was of critical importance. We offered a supervised exercise program that would provide several individual benefits including less treatment-related side-effects, better quality of life, and improved social well-being.

A second important step is to motivate patients for continuous adherence to the exercise program during the study period. Rates of adherence were reported in the literature varying widely between 83 % [2] for an 18-week exercise intervention during breast cancer treatment, and 46 % for resistance training in another trial [8]. Recently, web-based programmes that target changes in exercise and dietary behaviors have been reported as being particularly useful [9] to maintain adherence. Among our patients, monthly evaluation of body composition, dietary consultation when requested and discussion of the individual results proved particularly useful. For patients not willing to participate in supervised high-intensity programmes, obviously home-based low-intensity physical activity programmes were identified as viable alternative being almost as effective in breast cancer patients [10].

Among the numerous outcome measures of randomized supervised exercise trials in cancer patients, cancer-related fatigue (CRF) is clearly one of the best documented endpoints [11, 12]. A recent meta-analysis identified nine high-quality studies that had included a total of 1156 patients and compared the impact of supervised aerobic exercise of various intensity levels to usual care [1]. Exercise interventions had a mean length of 21.4 weeks (SD 15.8) with a mean duration of 44 min (SD 15.2) and an average of 2.5 (SD 0.7) sessions per week. Cancer-related fatigue was significantly improved in the intervention group with a standardized mean difference of −0.51 (95 % CI, −0-81 to −0.21). With a length of 52 weeks, a usual duration of one hour and three sessions per week, exercise interventions in our trial were at the upper limit of the data provided by the metaanalysis.

Another well documented outcome measure in trials evaluating a possible impact of aerobic exercise in cancer patients is the peak oxygen consumption (VO_2 peak)_ during cardiopulmonary exercise testing (CPET). We have demonstrated in this study that VO_2 peak_ among 36 cancer patients increased by 1.7 ml/min/kg at 3 months and 1.1 ml/min/kg at 12 months, respectively, following a supervised aerobic exercise programme of two or three weekly sessions over a time period of one year. This increase seems particularly remarkable given the fact that over 80 % of the patients had an ongoing chemotherapy and/or radiation during the study period. Randomized trials from the literature that compared patients of interventional groups to those of control groups usually reported a decrease of VO_2 peak_ in the control groups between 1.02 ml/min/kg [13], and 1.31 ml/min/kg [8]. Courneya et al. [14] observed a difference in VO_2 peak_ in patients belonging to the interventional group vs the control group of only 0.98 ml/min/kg. This difference was explained by the stabilization of VO_2 peak_ in the interventional group and a decreased value in the control group. Subgroup analysis according to our data revealed a trend for a possible benefit in terms of oxygen consumption particularly among breast cancer patients (*p* = 0.11) and patient treated with curative intent (*p* = 0.001).

Another possible outcome measure for the effects of aerobic exercise in cancer patients may be represented by improvements in body composition. This pilot study was able to demonstrate a significant reduction in body mass index and body fat mass among 37 cancer patients. As compared with baseline, median body mass index decreased from 27.4 ± 7.2 kg/m^2^ to 25.9 ± 7.0 kg/m^2^ and 26.9 ± 6.7 kg/m^2^ at 3 (*p* = 0.001) and 12 months (*p* = 0.015), respectively. Starting from a median value of 30.7 ± 15.4 kg for body fat mass, the patients reached 28.9 ± 15 kg at 3 months and 29.5 ± 13 kg at 12 months, respectively. Subgroup analysis from our data showed that mean BMI decreased by 1.3 ± 1.8 kg/m^2^ only in the group of younger patients at 3 months whereas in older patients the change was only −0.17 ± 1.3 kg/m^2^. This difference reached statistical significance (*p* = 0.03).

Similar data have been presented by van den Dungen et al. [4] and O’Neill et al. [15]. This latter group randomized a total of 94 prostate cancer patients receiving androgen deprivation therapy to an interventional arm that consisted of dietary and physical activity recommendation versus usual care. The primary outcome of interest was body composition. The interventional group had a significant (*p* < 0.001) reduction in weight, body mass index and percentage fat mass compared to the control group at six months; the between-group differences were −3.3 kg, −1.1 kg/m^2^ and −2.1 %, respectively.

## Conclusion

In conclusion, an aerobic exercise programme in cancer patients with 2–3 weekly supervised sessions over 12 months was well feasible and demonstrated measurable improvement of oxygen consumption, body composition and quality of life. Further randomized prospective data in a larger patient population preferring breast cancer patients will be collected comparing the impact of one versus three months supervision.

## Additional file

Additional file 1: Figure S1. Development of body fat mass (in %) among 37 cancer patients at baseline and following 1,2,3,6 and 12 months of continuous physical exercise. Figure S2 Quality of life data on symptom scales for 37 cancer patients at baseline and at 3 and 12 months following continuous physical exercise. Figure S3 Global health status in 37 cancer patients at baseline and at 3 and 12 months following continuous physical exercise. Table S1 Data for body mass composition (median values ± SD) among 39 cancer patients at baseline and at 1,2,3,6 and 12 months following physical exercise. FM, Fat mass; BCM, body cell mass; BMI, body mass index. RARF PA, phase angle. Table S2 Peak oxygen consumption (VO_2max_) among 36 cancer patients at baseline and after 3 and 12 months during continuous physical exercise (median values ± SD). Table S3 Functional scales among 39 cancer patients at baseline and after 3 and 12 months during continuous physical exercise (median values ± SD). Table S4 Symptom-scales among 39 cancer patients at baseline and after 3 and 12 months during continuous physical exercise (median values ± SD). Table S5 Subgroup analysis for the BMI difference at 3 months vs. baseline and 12 months vs. Baseline. Table S6 Subgroup analysis for the fat mass difference at 3 months vs. baseline and 12 months vs. baseline. Table S7 Subgroup analysis for the difference of peak oxygen consumption at 3 months vs. baseline and 12 months vs. baseline. Table S8 Subgroup analysis for the difference of the global health status at 3 months vs. baseline and 12 months vs. Baseline. (PDF 291 kb)
