# Supplementary material for: Feasibility of a 12-month-exercise intervention during and after radiation and chemotherapy in cancer patients: impact on quality of life, peak oxygen consumption, and body composition
Source: Radiat Oncol. 2016 Mar 16;11:42. doi: 10.1186/s13014-016-0619-5 (PMC4793737; doi:10.1186/s13014-016-0619-5)
Supplement: Additional file 1: Figure S1. — Development of body fat mass (in %) among 37 cancer patients at baseline and following 1,2,3,6 and 12 months of continuous physical exercise. Figure S2 Quality of life data on symptom scales for 37 cancer patients at baseline and at 3 and 12 months following continuous physical exercise. Figure S3 Global health status in 37 cancer patients at baseline and at 3 and 12 months following continuous physical exercise. Table S1 Data for body mass composition (median values ± SD) among 39 cancer patients at baseline and at 1,2,3,6 and 12 months following physical exercise. FM, Fat mass; BCM, body cell mass; BMI, body mass index. RARF PA, phase angle. Table S2 Peak oxygen consumption (VO2max) among 36 cancer patients at baseline and after 3 and 12 months during continuous physical exercise (median values ± SD). Table S3 Functional scales among 39 cancer patients at baseline and after 3 and 12 months during continuous physical exercise (median values ± SD). Table S4 Symptom-scales among 39 cancer patients at baseline and after 3 and 12 months during continuous physical exercise (median values ± SD). Table S5 Subgroup analysis for the BMI difference at 3 months vs. baseline and 12 months vs. Baseline. Table S6 Subgroup analysis for the fat mass difference at 3 months vs. baseline and 12 months vs. baseline. Table S7 Subgroup analysis for the difference of peak oxygen consumption at 3 months vs. baseline and 12 months vs. baseline. Table S8 Subgroup analysis for the difference of the global health status at 3 months vs. baseline and 12 months vs. Baseline. (PDF 291 kb) [file 13014_2016_619_MOESM1_ESM.pdf]

## Supplementary Material:

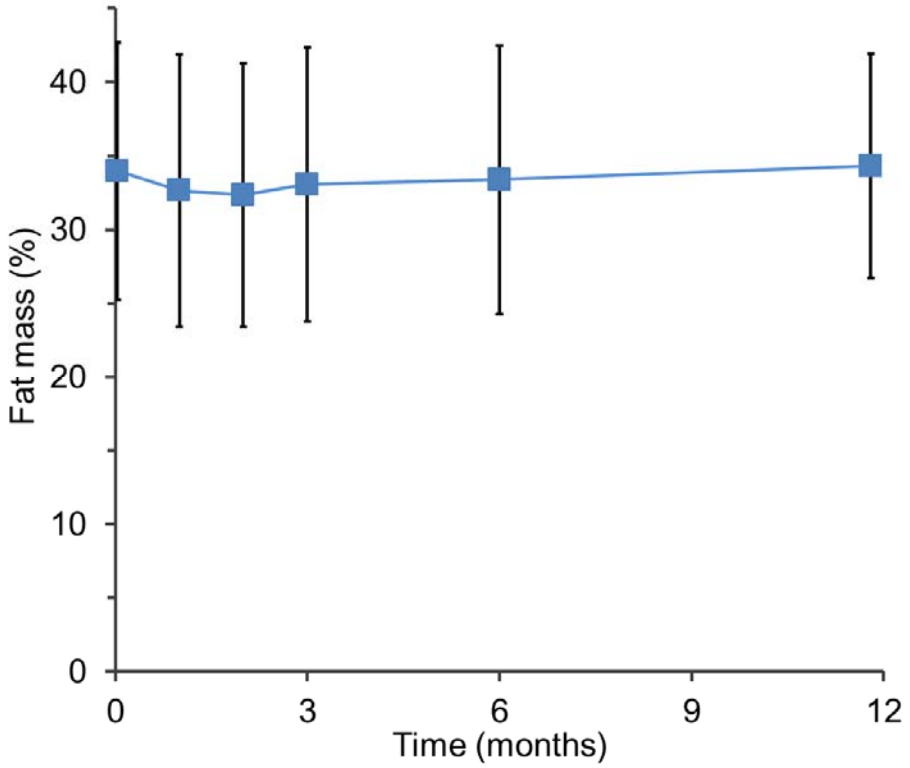


Supplementary Figure 1:
Development of body fat mass (in %) among 37 cancer patients at baseline and following 1, 2, 3, 6 and 12 months of continuous physical exercise.

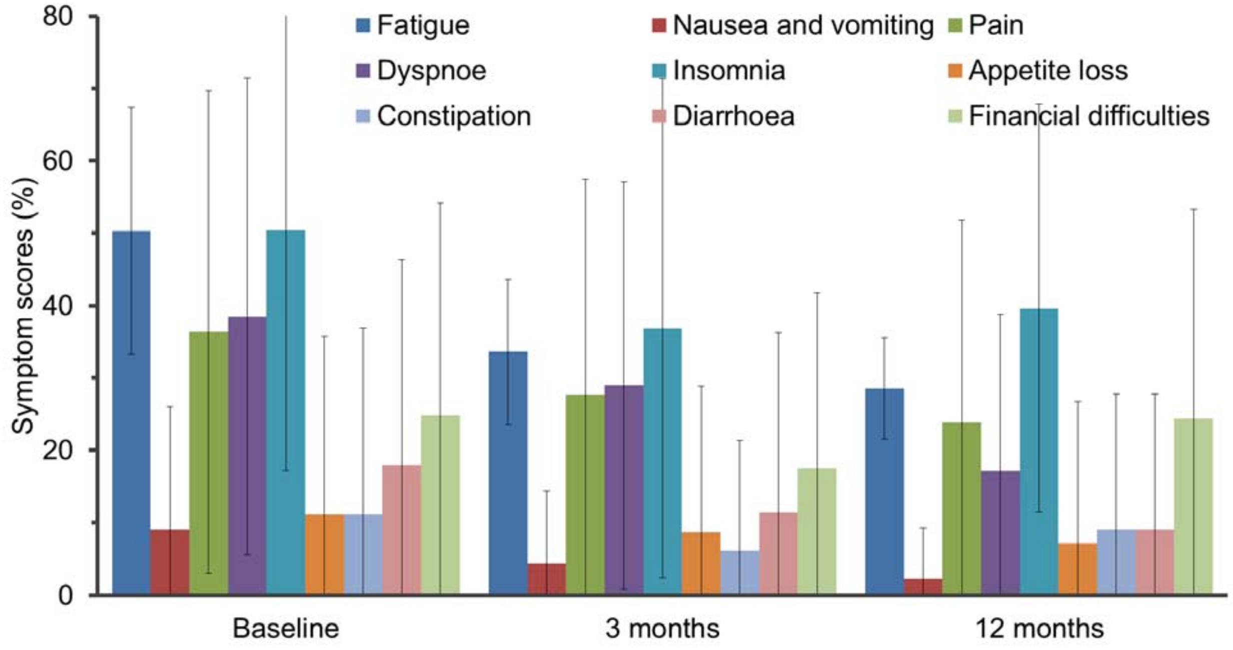


Supplementary Figure 2:
Quality of life data on symptom scales for 37 cancer patients at baseline and at 3 and 12 months following continous physical exercise

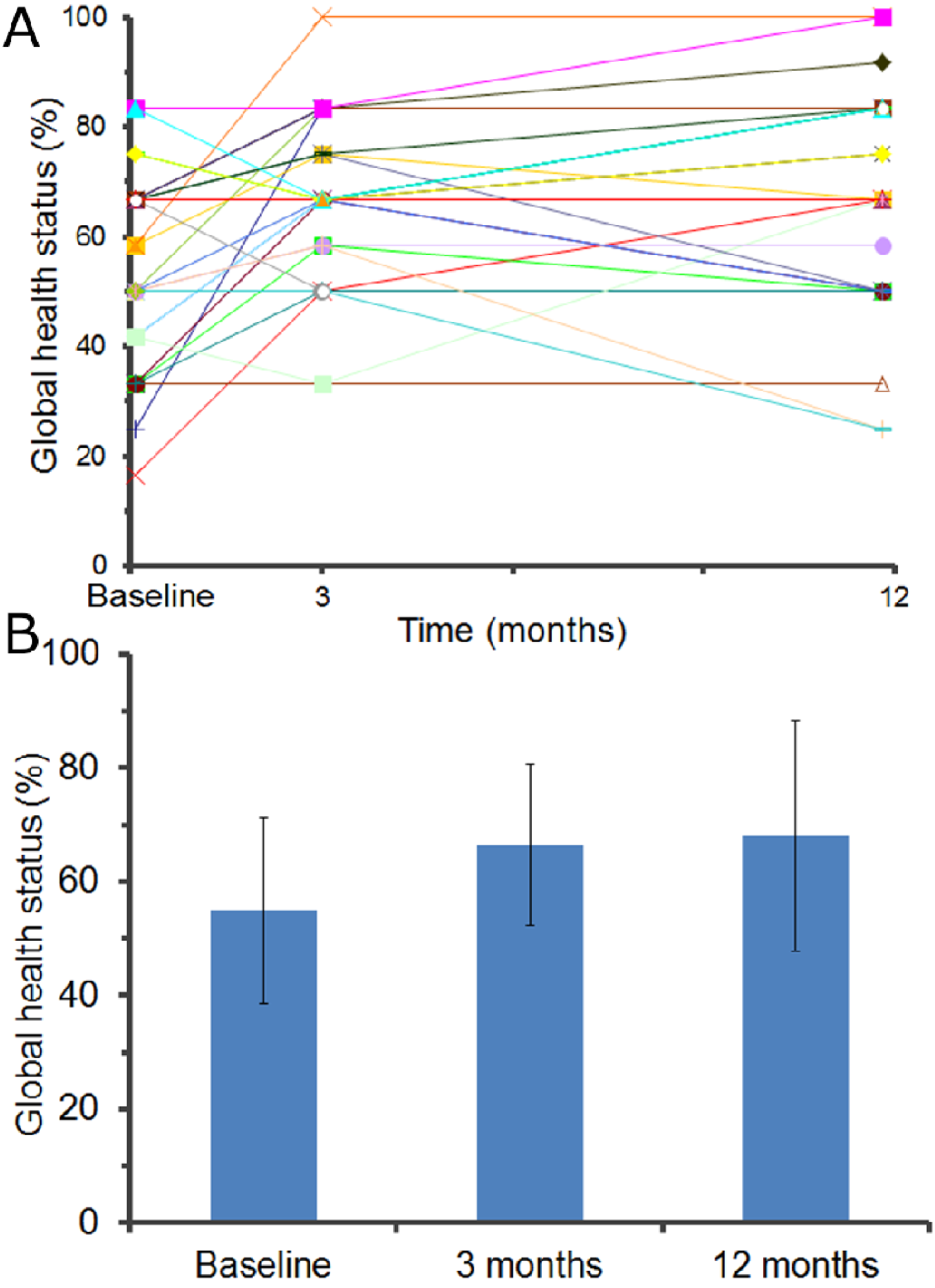


Supplementary Figure 3: Global health status in 37 cancer patients at baseline and at 3 and 12 months following continous physical exercise. A) Individual values and B) mean values. Error bars represents standard deviation.

Supplementary table 1:

Data for body mass composition (median values ±SD) among 39 cancer patients at baseline and at 1,2,3,6 and 12 months following physical exercise. FM, Fat mass; BCM, body cell mass; BMI, body mass index. RARF PA, phase angle.

| | Phase angle | FM % | FM kg | BCM kg | BMI |
|---|---|---|---|---|---|
| **0 months (n = 39)** | 5,39 (0,81) | 35,9 (8,8) | 30,7 (15,4) | 24,2 (4,0) | 28,9 (7,2) |
| **1 months (n = 35)** | 5,33 (0,71) | 34,9 (9,0) | 29,2 (15,5) | 23,6 (3,6) | 28,1 (7,3) |
| **2 months (n = 35)** | 5,36 (0,66) | 35,1 (8,7) | 29,4 (15,0) | 23,8 (3,5) | 28,2 (7,3) |
| **3 months (n = 39)** | 5,26 (0,58) | 34,6 (9,0) | 28,9 (14,6) | 23,8 (3,5) | 28,2 (7,0) |
| **6 months (n = 37)** | 5,26 (0,63) | 34,8 (8,7) | 29,2 (13,8) | 23,8 (3,0) | 28,5 (6,8) |
| **12 months (n = 38)** | 5,40 (0,75) | 35,3 (7,9) | 29,5 (13,3) | 24,0 (3,0) | 28,5 (6,7) |

Supplementary table 2:

Peak oxygen consumption ($VO_{2max}$) among 36 cancer patients at baseline and after 3 and 12 months during continuous physical exercise (median values ±SD).

| | **$VO_{2max}$ ml/min (SD)** | **$VO_{2max}$ ml/min per kg BW (SD)** |
|---|---|---|
| **Baseline** (n = 36) | 1421.0 (387.6) | 18.5 (5.6) |
| **3 months** (n = 36) | 1537.6 (300.2) (p = 0.006) | 20.4 (4.6) (p = 0.005) |
| **12 months** (n = 32) | 1520.1 (334.3) (p = 0.017) | 20.0 (4.7) (p = 0.003) |

Supplementary table 3:

Functional scales among 39 cancer patients at baseline and after 3 and 12 months during continuous physical exercise (median values ±SD).

| | **Physical functioning** | **Role functioning** | **Emotional functioning** | **Cognitive functioning** | **Social functioning** |
|---|---|---|---|---|---|
| **0 months** (n = 39) | 77,3 (18,3) | 55,6 (26,9) | 52,4 (24,5) | 66,7 (27,3) | 58,1 (28,8) |
| **3 months** (n = 38) | 83,9 (18,5) | 73,7 (26,7) | 63,4 (20,7) | 70,2 (30,5) | 72,4 (25,2) |
| **12 months** (n = 37) | 84,7 (21,3) | 76,1 (30,3) | 67,1 (23,8) | 76,1 (23,7) | 73,4 (27,3) |

Supplementary table 4:

Symptom-scales among 39 cancer patients at baseline and after 3 and 12 months during continuous physical exercise (median values ±SD).

| | **Fatigue** | **Nausea and vomiting** | **Pain** | **Dyspnoea** | **Insomnia** | **Appetite loss** | **Constipation** | **Diarrhoea** | **Financial difficulties** |
|---|---|---|---|---|---|---|---|---|---|
| **0 months** (n = 39) | 50,3 (27,4) | 9,0 (17,0) | 36,3 (33,3) | 38,5 (32,9) | 50,4 (33,2) | 11,1 (24,6) | 11,1 (25,7) | 17,9 (28,5) | 24,8 (29,3) |
| **3 months** (n = 38) | 33,6 (24,7) | 4,4 (10,0) | 27,6 (29,8) | 28,9 (28,1) | 36,8 (34,5) | 8,8 (20,0) | 6,1 (15,2) | 11,4 (24,8) | 17,5 (24,2) |
| **12 months** (n = 37) | 28,5 (23,8) | 2,3 (7,0) | 23,9 (27,9) | 17,1 (21,7) | 39,6 (28,2) | 7,2 (19,5) | 9,0 (18,7) | 9,0 (18,7) | 24,3 (29,0) |

Supplementary Table 5: Subgroup analysis for the BMI difference at 3 months vs. baseline and 12 months vs. Baseline

| patient characteristic | number of patients | mean value in kg/m² | standard deviation in kg/m² | p value |
|---|---|---|---|---|
| **BMI difference 3 months vs. Baseline** | | | | |
| ≤ 50 years | 18 | -1.31 | 1.83 | 0.031 |
| > 50 years | 21 | -0.18 | 1.31 | |
| men | 4 | -0.27 | 0.29 | 0.590 |
| women | 35 | -0.75 | 1.73 | |
| others | 13 | -0.97 | 1.97 | 0.470 |
| breast cancer | 26 | -0.56 | 1.49 | |
| curative | 32 | -0.72 | 1.80 | 0.855 |
| palliative | 7 | -0.59 | 0.71 | |
| **BMI difference 12 months vs. Baseline** | | | | |
| ≤ 50 years | 18 | -1.30 | 2.40 | 0.054 |
| > 50 years | 20 | -0.01 | 1.54 | |
| men | 4 | -0.35 | 1.19 | 0.787 |
| women | 34 | -0.65 | 2.16 | |
| others | 12 | -1.06 | 2.14 | 0.375 |
| breast cancer | 26 | -0.41 | 2.05 | |
| curative | 32 | -0.56 | 2.15 | 0.702 |
| palliative | 6 | -0.92 | 1.73 | |

Supplementary Table 6: Subgroup analysis for the fat mass difference at 3 months vs. baseline and 12 months vs. baseline

| patient characteristic | Number of patients | mean value in kg | standard deviation in kg | p value |
|---|---|---|---|---|
| **FM difference 3 months vs. Baseline** | | | | |
| ≤ 50 years | 18 | -2.9 | 3.5 | 0.037 |
| > 50 years | 21 | -0.8 | 2.3 | |
| men | 4 | -0.6 | 1.9 | 0.424 |
| women | 35 | -1.9 | 3.2 | |
| others | 13 | -2.6 | 3.5 | 0.264 |
| breast cancer | 26 | -1.4 | 2.8 | |
| curative | 32 | -1.8 | 3.3 | 0.901 |
| palliative | 7 | -1.6 | 1.5 | |
| **FM difference 12 months vs. Baseline** | | | | |
| ≤ 50 years | 18 | -3.0 | 5.0 | 0.071 |
| > 50 years | 20 | -0.4 | 3.3 | |
| men | 4 | -0.2 | 3.1 | 0.493 |
| women | 34 | -1.8 | 4.5 | |
| others | 12 | -2.7 | 4.2 | 0.316 |
| breast cancer | 26 | -1.1 | 4.4 | |
| curative | 32 | -1.5 | 4.5 | 0.677 |
| palliative | 6 | -2.3 | 3.9 | |

Supplementary Table 7: Subgroup analysis for the difference of peak oxygen consumption at 3 months vs. baseline and 12 months vs. baseline

| **Difference of peak oxygen consumption at 3 months vs. Baseline** | | | | |
|---|---|---|---|---|
| patient characteristic | number of patients | mean value in ml/min | standard deviation in ml/min | p value |
| ≤ 50 years | 17 | 121.1 | 305.9 | 0.926 |
| > 50 years | 19 | 112.6 | 235.5 | |
| men | 4 | -229.3 | 404.0 | 0.004 |
| women | 32 | 159.8 | 217.6 | |
| others | 11 | 8.9 | 344.1 | 0.109 |
| breast cancer | 25 | 164.0 | 216.8 | |
| curative | 30 | 177.3 | 215.8 | 0.001 |
| palliative | 6 | -187.0 | 310.6 | |
| **Difference of peak oxygen consumption at 12 months vs. baseline** | | | | |
| ≤ 50 years | 16 | 84.9 | 242.8 | 0.507 |
| > 50 years | 16 | 152.3 | 318.6 | |
| men | 2 | 184.5 | 385.4 | 0.738 |
| women | 30 | 114.2 | 280.7 | |
| others | 8 | 51.9 | 330.8 | 0.447 |
| breast cancer | 24 | 140.8 | 266.1 | |
| curative | 30 | 130.4 | 277.2 | 0.365 |
| palliative | 2 | -58.5 | 384.0 | |

Supplementary Table 8: Subgroup analysis for the difference of the global health status at 3 months vs. baseline and 12 months vs. baseline

| **Difference of global health status after 3 months vs baseline** | | | | |
|---|---|---|---|---|
| patient characteristic | number of patients | mean value in % | standard deviation in % | p value |
| ≤ 50 years | 15 | 10.6 | 17.7 | 0.833 |
| > 50 years | 21 | 11.9 | 19.5 | |
| Men | 4 | -4.2 | 10.8 | 0.075 |
| Women | 32 | 13.3 | 18.4 | |
| Others | 12 | 0.7 | 16.5 | 0.012 |
| breast cancer | 24 | 16.7 | 17.4 | |
| Curative | 30 | 12.5 | 18.0 | 0.409 |
| Palliative | 6 | 5.6 | 21.5 | |
| **Difference of global health status after 12 months vs baseline** | | | | |
| ≤ 50 years | 15 | 15.6 | 24.0 | 0.561 |
| > 50 years | 20 | 10.8 | 23.3 | |
| men | 3 | 2.8 | 4.8 | 0.442 |
| women | 32 | 13.8 | 24.2 | |
| others | 10 | -1.7 | 25.4 | 0.017 |
| breast cancer | 25 | 18.7 | 20.2 | |
| curative | 31 | 15.1 | 20.8 | 0.123 |
| palliative | 4 | -4.2 | 37.6 | |
